# Dupilumab in patients with bullous pemphigoid and concomitant atopy

**DOI:** 10.3389/fphar.2025.1656089

**Published:** 2025-08-29

**Authors:** Katerina Jobst, Stephan R. Künzel, Stefan Beissert, Susanne Abraham

**Affiliations:** ^1^ Department of Dermatology, Faculty of Medicine Carl Gustav Carus, University Allergy Center, Technische Universität Dresden, Dresden, Germany; ^2^ Institute for Transfusion Medicine, Faculty of Medicine Carl Gustav Carus, Technische Universität Dresden, Dresden, Germany; ^3^ DRK Blutspendedienst Nord-Ost gGmbH, Department of Experimental Transfusion Medicine, Dresden, Germany

**Keywords:** bullous pemphigoid, atopic dermatitis, dupilumab, tralokinumab, Anti-IL4/13 antibody, targeted therapy, drug repurposing, atopy

## Abstract

**Background:**

Bullous pemphigoid (BP) and atopic dermatitis (AD) are chronic inflammatory skin diseases that may share overlapping immunopathogenic mechanisms, particularly a type 2 immune response. Emerging evidence suggests that dupilumab, an IL-4Rα antagonist, may be effective in treating both conditions.

**Methods:**

In this retrospective case series, twelve patients (mean age: 78.6 years; range: 67–93 years) with moderate to severe BP and a history of AD were included. All patients received dupilumab and were monitored over 12 weeks. Clinical activity was assessed using BPDAI scores, pruritus NRS, and DLQI.

**Results:**

At week 12, complete remission of bullous skin lesions was observed in all patients (100%), with 83.3% already achieving this by week 4. Pruritus improved significantly (p < 0.0001), with 58.3% achieving complete resolution (NRS 0/10) at week 12. Notably, two patients had previously received tralokinumab without clinical improvement, underscoring the distinct efficacy of dupilumab in this setting. Patient satisfaction was uniformly high (score 5/5). No adverse events were reported. Dupilumab was well tolerated, even in elderly, multimorbid patients.

**Conclusion:**

Dupilumab appears to be a safe and highly effective treatment for patients with concurrent BP and AD, leading to rapid and sustained symptom control, resolution of skin lesions, and high patient satisfaction.

## Introduction

Bullous pemphigoid (BP) is the most common autoimmune bullous disease characterized by subepidermal blistering, typically affecting elderly patients (Kridin and Ludwig, 2018). Pathogenetically, an autoimmune T cell response and the production of IgG and IgE autoantibodies against the hemidesmosomal proteins BP180 and BP230 play a crucial role (Kasperkiewicz and Zillikens, 2007; Schmidt and Zillikens, 2013; Miyamoto et al., 2019). Most patients have autoantibodies directed against the extracellular domain of the hemidesmosomal protein BP180, while the intracellular protein BP230 is less commonly targeted. The presence of these autoantibodies leads to neutrophil chemotaxis and detachment at the dermo-epidermal junction (Kasperkiewicz and Zillikens, 2007; Schmidt and Zillikens, 2013; Miyamoto et al., 2019).

Typical lesions include tense blisters on urticarial or erythematous bases associated with severe pruritus. Isolated itch without skin lesions can be present in the premonitory stage of the disease (Kasperkiewicz and Zillikens, 2007; Schmidt and Zillikens, 2013; Miyamoto et al., 2019). Diagnostic procedures encompass histopathological evaluation, detection of IgG and/or C3 deposits at the basal membrane zone via direct immunofluorescent microscopy, presence of autoantibodies in patient sera in indirect immunofluorescent microscopy, and quantification of circulating autoantibodies against BP180 and/or BP230 via ELISA (Kridin and Ludwig, 2018; Miyamoto et al., 2019).

Therapeutic approaches vary from topical corticosteroid (TCS) therapy to various systemic treatments depending on the patient’s clinical status and disease severity. Due to the chronic nature of the disease and the frequent comorbidities in elderly patients, long-term management is usually required, necessitating careful consideration of potential side effects and the feasibility of treatment options in the home setting (Holtsche et al., 2023; Zhao et al., 2023; Karakioulaki et al., 2024; Murrell et al., 2024).

Currently, numerous new and promising therapeutic modalities are under investigation. Multiple agents have already been tested as part of clinical phase III trials, such as bertilimumab (eotaxin-1 [CCL1] inhibitor), benralizumab (IL-5Rα inhibitor), dupilumab (IL-4αR inhibitor), nomacopan (inhibitor of C5a/LTB4), and efgartigimod (inhibitor of neonatal FcR). Furthermore, avdoralimab (C5aR1 inhibitor), tildrakizumab (IL-23 inhibitor), and sutimlimab (C1 inhibitor) are currently in the earlier stages of investigation. Other agents, such as omalizumab (Cε3 domain of IgE inhibitor) or upadacitinib (JAK-1 inhibitor), have been successfully used in individual cases (Holtsche et al., 2023; Zhao et al., 2023; Karakioulaki et al., 2024; Murrell et al., 2024).

This growing body of therapeutic research includes the concept of drug repurposing, in which agents approved for one disease are investigated for efficacy in other indications. Drug repurposing can accelerate clinical translation by leveraging existing safety and pharmacokinetic data, which is especially relevant for rare or difficult-to-treat diseases such as BP (Nosengo, 2016; Pushpakom et al., 2019). While the repurposing of small molecules is well established, the use of monoclonal antibodies (mAbs) in this context is relatively novel, particularly in dermatology. Dupilumab has been approved for the treatment of moderate to severe atopic dermatitis (AD) since the end of 2017 and subsequently also for other indications. Its favorable safety profile, particularly in elderly and multimorbid patients, makes it an attractive candidate for repurposing in BP (Blauvelt et al., 2017; Patruno et al., 2021).

In a case series involving 13 patients with BP treated with dupilumab, 92.3% (12 out of 13) responded to the therapy (Abdat et al., 2020). Among these, 53.8% (7 out of 13) achieved complete remission. This study suggests that dupilumab is also an effective treatment for BP, a finding that has also been supported by systematic reviews and meta-analyses confirming its potential efficacy (Da Silva et al., 2025; Mainville et al., 2025). Currently, a study involving 10 patients in China with diverse comorbidities has also provided evidence of the effectiveness of this therapy in a 1-year follow-up trial (Wang et al., 2023). In addition, the pivotal approval study evaluating dupilumab in patients with BP is currently being conducted (Murrell et al., 2024).

### Context

In the present analysis, we aimed to demonstrate both the treatment response and favorable tolerability of dupilumab in patients with confirmed BP and an atopic predisposition. Our findings support the potential repurposing of a therapy already approved for AD for the treatment of BP.

### Materials and methods

This case series of 12 patients investigates the occurrence of AD in patients concurrently diagnosed with BP and treated with dupilumab.

Diagnosis of BP was confirmed through direct immunofluorescence (DIF) and indirect immunofluorescence (IIF), with all patients exhibiting BP-180-antibodies, and about half of the patients showing both BP-180 and BP-230 antibodies.

AD was diagnosed by the diagnostic criteria according to Hanifin and Rajka (1980) and the severity was evaluated using Eczema Area and Severity Index (EASI) and SCORing Atopic Dermatitis (SCORAD) scoring systems. The Erlangen atopy score was at least 10 points in all patients. BP severity was assessed using the Bullous Pemphigoid Disease Area Index (BPDAI). Furthermore, the pruritus intensity was assessed using the Peak *Pruritus* Numerical Rating Scale (*NRS*).

The patients‘ satisfaction with the treatment outcome was evaluated on the scale of 0–5 (0 indicating not satisfied at all, 5 indicating very satisfied with the treatment).

Dupilumab was administered according to the approved dosing regimen for AD: an initial loading dose of 600 mg subcutaneously, followed by 300 mg every 2 weeks. Dose adjustments were not made during the treatment period.

Statistical Analysis: Graphic data representation and statistical analysis were performed using graph pad prism 10 (GraphPad Software; Boston, United States). Comparisons between two groups were performed using a two-sided, unpaired t-test with Welch’s correction. One-Way ANOVA followed by Tukey post-test was used for comparisons between three groups. P-values <0.05 were considered statistically significant.

Limitations of this study include the small sample size (n = 12) and the potential for selection bias. Furthermore, the generalizability of the findings may be restricted due to the specific characteristics of the patient population. While atopic predisposition was assessed using the Erlangen Atopy Score, distinguishing atopic eczema from BP lesions remains challenging. The advanced age of several patients also posed limitations, as it impeded consistent follow-up and, in some cases, the reliable evaluation of quality of life.

## Results

The study included 12 patients (n = 12) with a mean age of 78.6 years (range: 67–93 years), comprising 7 men and 5 women. Demographic characteristics of the patient population are summarized in Table 1.

**TABLE 1:** Demographic characteristics.

Patient ID	Age	Sex	ECOG Score	BMI [kg/m2]	GFR [mL/min/1,73]	ALT/AST/GGT [µmol/(s*L)]	Cardiovascular and metabolic diseases	History of cancer
#1	82	F	1	28.8	70	0.29/1.08/0.68	Hypertension	None
#2	83	M	1	26.4	53	0.60/0.51/0.74	art HT, T2D, HLP, Hyperuricemia	None
#3	69	M	0	29.4	90	0.35/0.33/0.54	art HT, Hyperlipidemia, BPH	None
#4	79	M	1	29.1	59	0.25/0.23/0.26	art HT, NAFLD, BPH	Basal-cell carcinoma
#5	72	F	2	35.9	62	0.59/1.01/4.29	art HT, COPD, Nephrotic Syndrome	None
#6	84	M	1	21.3	54	0.49/0.51/0.55	art HT, Atrial Fibrillation, T2D, HLP	None
#7	79	M	2	33.0	54	0.11/0.17/0.26	art HT, NSTEMI, T2D	None
#8	67	F	2	39.5	40	0.51/0.67/2.85	art HT, Heart Failure (NYHA III), T2D, HLP	None
#9	85	M	1	23.5	66	0.32/0.47/0.52	Asthma bronchiale	Metastasized Kidney Carcinoma
#10	84	F	1	24.1	80	0.14/0.24/0.34	art HT, T2D	None
#11	66	F	4	25.4	90	0.23/0.35. 0.49	art HT	None
#12	93	M	0	24.8	20	0.26/0.20/0.66	art HT, DM Typ 2, CKD	Prostate Carcinoma

Legend: ECOG, eastern cooperative oncology group performance status; BMI, body mass index; GFR, glomerular filtration rate; ALAT, Alanine Aminotransferase (ALT); ASAT, Aspartate Aminotransferase (AST); GGT, Gamma-Glutamyl Transferase; T2D = Type 2 Diabetes Mellitus; HLP, hyperlipidemia; COPD, chronic obstructive pulmonary disease; art HT, arterial hypertension; CKD, chronic kidney disease.

An overview of BP-specific laboratory and clinical parameters is provided in Table 2. All patients had detectable autoantibodies against BP180, while approximately half also had antibodies against BP230. Anti-BP180 ELISA values varied widely, with some patients showing markedly elevated titers (up to over 6800 RE/mL). All patients exhibited elevated total IgE levels and, in some cases, pronounced eosinophilia, indicating a strong type 2 immune response. All patients presented with moderate to severe cutaneous activity of BP, without mucosal involvement. Pruritus was a consistent symptom in all cases, and all patients reported a reduced quality of life as reflected by elevated Dermatology Life Quality Index (DLQI) scores. Half of the patients received oral prednisone at the start of treatment with dupilumab, as detailed in Table 2. Prednisone dosages varied between 20 mg and 50 mg daily and were tapered before or shortly after initiating dupilumab. Importantly, all patients were off systemic corticosteroids by week 12.

**TABLE 2:** Laboratory and clinical baseline parameters.

Pat. ID	ELISA Anti-BP180 [RE/mL]	ELISA Anti-BP230 [RE/mL]	Total IgE [mU/L]	Eosinophils [absolute/relative]	BPDAI [Activity/Damage]	SCORAD	oSCORAD	Erlangen score	EASI	DLQI [x/30]	Previous therapy	Oral prednisone (initial dose)
#1	55.9	0	1871	1.0/16.2%	A 54/D 2	51.51	34.51	19.5	17.9	24	none	none
#2	1508.2	186.8	4870	5.29/30.9%	A 45/D7	65.54	48.54	15.5	11.2	12	none	none
#3	1000.3	0	2693	0.21/1.5%	A38/D1	44.64	34.64	10.5	18.3	4	none	20 mg
#4	259.7	98.8	364	1.28/14.9%	A71/D1	61.85	46.85	15.5	23.1	10	none	50 mg
#5	1064.8	787.6	958	0.75/5.2%	A62/D2	58.41	47.41	11	20.7	11	none	none
#6	3298.6	0	516	0.75/4.7%	A46/D7	53.87	41.87	18	26	16	none	none
#7	71.7	0	644	3.5/27.2%	A31/D0	40.24	33.24	13	N.A	7	none	none
#8	1900.7	1248	6489	2.73/29.7%	A61/D2	55.49	49.49	12.50	32.5	4	none	50 mg
#9	70.6	23.1	116	0.37/5.9%	A27/D0	61.9	24.0	10.5	24.0	27	Dapson 3 months. 1 cycle IVIG	none
#10	510.4	0	306	0.82/6.4%	A66/D7	43.5	16.0	14.0	16.0	22	none	30 mg
#11	118.7	89.1	428	0.05/0.7%	A124/D11	46.1	29.8	11.5	29.8	N.A.	Tralokinumab (3 m)	30 mg
#12	6807.7	0	1297	8.71/34.1%	A49/D0	40.15	17.1	15.0	17.1	16	Doxycycline (3 m); Tralokinumab (3 m)	30 mg

Legend: BP, Antibodies = Bullous pemphigoid-specific autoantibodies; ELISA, Anti-BP180 = Antibodies against BP180 (type XVII, collagen), measured by enzyme-linked immunosorbent assay (ELISA), in relative units per milliliter (RE/mL); ELISA, Anti-BP230 = Antibodies against BP230 (a hemidesmosomal intracellular protein), measured by ELISA, in RE/mL; Total IgE = Total immunoglobulin E levels; BPDAI [Activity/Damage] = Bullous Pemphigoid Disease Area Index, subdivided into activity and damage scores; SCORAD, scoring atopic dermatitis; Erlangen Atopy Score = Quantification of atopic diathesis based on personal and family history; EASI, eczema area and severity index; DLQI, Dermatology Life Quality Index (maximum score: 30); N.A., Not applicable due to the patient’s general condition.

**FIGURE 1:**
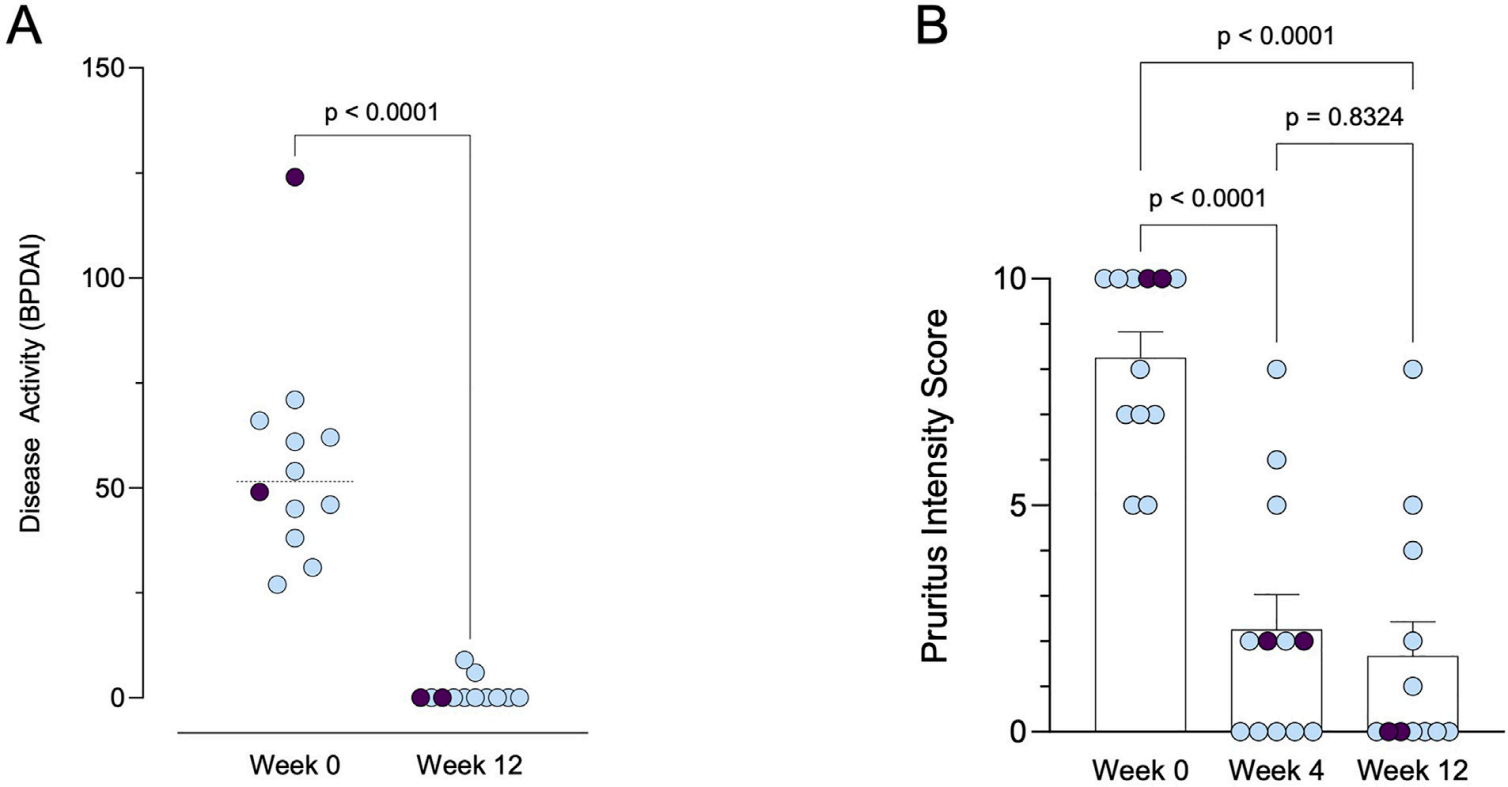
Clinical response to dupilumab therapy over time. **(A)** Disease activity measured by BPDAI (range: 0–240) at baseline and after 12 weeks of treatment. Patients with prior treatment using tralokinumab are indicated in purple. **(B)** Pruritus intensity scores (range: 0–10) at baseline, week 4, and week 12. Patients with prior treatment using tralokinumab are indicated in purple.

At 4 and 12 weeks following initiation of dupilumab treatment, all 12 patients demonstrated a positive response (Figures 1A,B; Table 3). Complete remission of bullous skin lesions was observed in 83.3% of patients (n = 10) at week 4, increasing to 100% (n = 12) by week 12. Pruritus improved in all patients (p < 0.0001, baseline vs week 12); however, one patient continued to report a pruritus intensity of 8 out of 10 after 12 weeks, despite complete healing of the skin. Complete resolution of pruritus symptoms (NRS = 0/10) was achieved in 58.3% (7/12) at week 12. It should be noted that the use of TCS was allowed throughout the treatment period and may have contributed to the observed symptom control in some cases. All of these patients also achieved complete clearance of bullous skin lesions (Figure 1A; p < 0.0001). Only two patients showed minimal residual activity with mild erythema visible on the BPDAI with 6 points (baseline value 54) and 9 points (baseline value 71), respectively.

**TABLE 3:** Outcome parameters.

Patient ID	Initial pruritus (NRS)	Pruritus after 4 weeks (NRS)	Pruritus after 12 weeks (NRS)	No blistering after	Patient satisfaction (0: low, 5 high)	Initial BPDAI [Activity]	BPDAI [Activity] after 12 weeks	Side effects
#1	10/10	0/10	0/10	4 weeks	5/5	A54	A6	none
#2	5/10	0/10	0/10	6 weeks	5/5	A45	A0	none
#3	7/10	0/10	0/10	4 weeks	5/5	A38	A0	none
#4	7/10	6/10	4/10	4 weeks	5/5	A71	A9	none
#5	10/10	8/10	8/10	2 weeks	5/5	A62	A0	none
#6	8/10	2/10	1/10	2 weeks	5/5	A46	A0	none
#7	7/10	0/10	0/10	2 weeks	5/5	A31	A0	none
#8	5/10	0/10	0/10	2 weeks	5/5	A61	A0	none
#9	10/10	5/10	5/10	4 weeks	5/5	A27	A0	none
#10	10/10	2/10	2/10	2 weeks	5/5	A66	A0	none
#11	10/10	2/10	0/10	4 weeks	5/5	A124	A0	none
#12	10/10	2/10	0/10	4 weeks	5/5	A49	A0	none

Legend: BPDAI [Activity] = Bullous Pemphigoid Disease Area Index; NRS: numeric rating scale.

Two patients had previously been treated with tralokinumab, which showed no clinical improvement after 3 months. Following a switch to dupilumab, both patients demonstrated a marked clinical improvement, with complete resolution of pruritus and no visible blister formation. No adverse effects associated with dupilumab therapy were reported. Patient satisfaction with the treatment was uniformly high, with all patients reporting a score of 5, indicating a high level of satisfaction, including patients without complete clearance of the skin lesions and less than 50% reduction in pruritus intensity. These findings suggest that dupilumab therapy effectively addresses both the primary and secondary objectives of treating concomitant AD and BP, with favorable outcomes in terms of symptom resolution and patient satisfaction.

## Discussion

BP poses a considerable challenge in dermatology, particularly given its increasing incidence and predilection for affecting elderly individuals (Kridin and Ludwig, 2018). The pathogenesis of this condition involves a complex interplay of autoimmune responses, characterised by the production of autoantibodies targeting hemidesmosomal proteins, primarily BP180 and less commonly BP230 (Schmidt and Zillikens, 2013; Miyamoto et al., 2019). These autoantibodies have been demonstrated to trigger inflammatory cascades, resulting in the formation of blisters at the dermo-epidermal junction (Kasperkiewicz and Zillikens, 2007). The clinical manifestations of BP, such as tense blisters over urticarial lesions accompanied by severe pruritus, impose substantial morbidity on patients (Schmidt and Zillikens, 2013; Miyamoto et al., 2019).

Diagnostic strategies for BP encompass histopathological examination, direct and indirect immunofluorescence microscopy, and serological testing for autoantibodies (Kridin and Ludwig, 2018; Miyamoto et al., 2019). Treatment modalities range from TCS to systemic agents, with therapeutic selection guided by disease severity and patient-specific factors (Karakioulaki et al., 2024). However, the chronic nature of BP necessitates long-term management, underscoring the importance of balancing efficacy with safety and patient convenience (Karakioulaki et al., 2024).

In recent years, the therapeutic landscape for BP has witnessed significant expansion, with several novel agents demonstrating promise in clinical trials. Among these, dupilumab—an interleukin-4 receptor alpha inhibitor—has emerged as a potential treatment option for BP (Holtsche et al., 2023; Karakioulaki et al., 2024).

Dupilumab can modulate chemokine-ligand 18, IL-4, and IL-13, which are Th2-related cytokines elevated in BP patients. These cytokines are found in higher levels in both sera and blister fluid of BP patients. They contribute to the maintenance of Th2-type responses, which are implicated in the loss of tolerance against the BP180 antigen. Thus, targeting these cytokines with dupilumab presents a novel therapeutic approach for treating cutaneous autoimmune bullous diseases (Russo et al., 2020; 2022; Maglie et al., 2024). Furthermore, the analysis of cytokine gene polymorphisms and their clinical relevance in Chinese patients with BP revealed elevated concentrations of IL-1β and IL-13 in the sera of BP patients compared to controls (Wang et al., 2022). The IL-13 genotype was notably associated with BP recurrence. These findings suggest that IL-13 plays a significant role in the pathogenesis of BP and could serve as a promising target for therapy and a prognostic marker. This highlights the potential utility of targeting IL-13 in the treatment of BP and underscores its significance as a prognostic indicator (Wang et al., 2022). In two of our patients, treatment with tralokinumab did not lead to clinical improvement; however, a rapid clinical response was observed following the initiation of dupilumab (Figures 1A,B; Table 3). Dupilumab inhibits both IL-4 and IL-13 signaling by blocking the IL-4 receptor alpha, whereas tralokinumab selectively targets IL-13. These findings suggest that IL-4–mediated pathways may play a critical role in the pathogenesis of BP, beyond the contribution of IL-13 alone.

Our study adds to the growing body of evidence supporting the efficacy and safety of dupilumab, especially in the patient population with concomitant AD. The results of our retrospective case series demonstrate notable improvements in both primary and secondary endpoints following dupilumab therapy. Complete remission of bullous skin lesions was achieved in the majority of patients, with a progressive increase in response rates over the 12-week observation period. Additionally, a significant reduction in pruritus symptoms was observed in all patients, with a substantial proportion achieving complete resolution (Table 3; Figure 1B). According to baseline characteristics, including SCORAD and EASI scores, the patients were initially classified as having moderate to severe AD. However, it should be noted that early stages of BP can clinically mimic eczematous lesions, potentially resembling AD. This diagnostic overlap may have influenced the initial classification and must be considered when interpreting baseline disease severity and treatment response.

In all our patients, TCS were used prior to initiating dupilumab therapy to manage inflammatory symptoms. During treatment with dupilumab, all patients were able to reduce or discontinue TCS use, with only occasional application of medium-potency corticosteroids in cases of localized flare-ups.

In the treatment of AD, dupilumab is generally considered a safe therapy with few adverse effects. However, in real-world settings, the incidence of the known side effect conjunctivitis appeared to be somewhat higher (up to 33.7%), although it was reported less frequently in older patients (Abraham et al., 2020; Zhang et al., 2024). In a study approximately 15% of elderly patients treated with dupilumab for AD experienced conjunctivitis (Patruno et al., 2021), 8% experienced dry eyes, and 6% had facial redness or erythema (Patruno et al., 2021). These adverse effects in elderly patients were generally mild and did not lead to discontinuation of the therapy (Patruno et al., 2021). Notably, in our patients no adverse effects related to dupilumab therapy were reported, highlighting its favorable safety profile.

The high level of patient satisfaction with dupilumab treatment underscores its potential as a valuable therapeutic option for BP in patients with concurrent AD. Despite the small sample size and retrospective design of our study, these findings contribute to the growing evidence supporting the use of dupilumab in this challenging patient population.

At the moment, a clinical phase II/III trial is being conducted (NCT04206553, LIBERTY-BP) and will elucidate the long-term efficacy and safety of dupilumab in BP management (Murrell et al., 2024). At the 2025 American Academy of Dermatology (AAD) Annual Meeting, late-breaking results from the LIBERTY-BP Phase 3 trial demonstrated that 20% of patients treated with dupilumab achieved sustained remission at week 36 compared to only 4% in the placebo group (*p* = 0.0114) (Sanofi, 2025). Approximately half of the patients in our investigation received oral prednisone at the start of treatment, as detailed in Table 2. Prednisone dosages ranged from 20 mg to 50 mg daily and were tapered before or shortly after the initiation of dupilumab. Notably, all patients were off systemic corticosteroids by week 12. This is of particular interest when compared to the LIBERTY-BP (NCT04206553) trial, in which systemic corticosteroids were tapered over a longer period with complete discontinuation targeted by week 16. Our findings may therefore further support the corticosteroid-sparing potential of dupilumab in BP and suggest that, in selected patients, systemic corticosteroids can be withdrawn even more rapidly under effective biologic therapy.

Eosinophils have been shown to play a key role in the development of BP. Dupilumab also proves good efficacy in other diseases in which there is an increase in eosinophils, such as eosinophilic esophagitis (Simon et al., 2017; Dellon et al., 2022). Given the limited treatment options available for this complex dermatological condition, dupilumab represents a promising addition to the therapeutic armamentarium for BP, offering the potential for improved disease control and enhanced patient quality of life. In addressing BP, there is a pressing demand for interventions characterized by favorable safety profiles and greater selectivity in targeting the immune system compared to the prevailing broad immunosuppressive approaches.

This retrospective case series supports the repurposing of dupilumab, originally approved for AD, as a safe and effective treatment option for BP, particularly in elderly and multimorbid patients. The favorable safety profile, combined with marked clinical improvements and high patient satisfaction, highlights dupilumab’s potential to address an important unmet need in the management of this challenging disease.

## Data Availability

The raw data supporting the conclusions of this article will be made available by the authors, without undue reservation.
